# Cancer cell enrichment on a centrifugal microfluidic platform using hydrodynamic and magnetophoretic techniques

**DOI:** 10.1038/s41598-021-81661-2

**Published:** 2021-01-21

**Authors:** Amir Shamloo, Amin Naghdloo, Mohsen Besanjideh

**Affiliations:** Department of Mechanical Engineering, Sharif University of Technology, Tehran, Iran

**Keywords:** Biotechnology, Engineering, Nanoscience and technology

## Abstract

Isolation of rare cancer cells is one of the important and valuable stages of cancer research. Regarding the rarity of cancer cells in blood samples, it is important to invent an efficient separation device for cell enrichment. In this study, two centrifugal microfluidic devices were designed and fabricated for the isolation of rare cancer cells. The first design (passive plan) employs a contraction–expansion array (CEA) microchannel which is connected to a bifurcation region. This device is able to isolate the target cells through inertial effects and bifurcation law. The second design (hybrid plan) also utilizes a CEA microchannel, but instead of using the bifurcation region, it is reinforced by a stack of two permanent magnets to capture the magnetically labeled target cells at the end of the microchannel. These designs were optimized by numerical simulations and tested experimentally for isolation of MCF-7 human breast cancer cells from the population of mouse fibroblast L929 cells. In order to use the hybrid design, magnetite nanoparticles were attached to the MCF-7 cells through specific Ep-CAM antibodies, and two permanent magnets of 0.34 T were utilized at the downstream of the CEA microchannel. These devices were tested at different disk rotational speeds and it was found that the passive design can isolate MCF-7 cells with a recovery rate of 76% for the rotational speed of 2100 rpm while its hybrid counterpart is able to separate the target cells with a recovery rate of 85% for the rotational speed of 1200 rpm. Although the hybrid design of separator has a better separation efficiency and higher purity, the passive one has no need for a time-consuming process of cell labeling, occupies less space on the disk, and does not impose additional costs and complexity.

## Introduction

Cell separation is a basic and essential stage of many biological investigations, especially in the field of single cell analysis^1^ and drug development^2^. In the last two decades, microfluidic technology has provided different techniques for separation and sorting various biological components based on their physical and chemical properties. Generally, these techniques are categorized into three main groups, including passive, active, and hybrid (combined) techniques^3^. Active methods utilize an external force field for cell separation and manipulation while the passive ones just rely on the interaction of particles with the flow field and the geometry of microchannels. Obviously, the hybrid methods are the combination of passive and active separation methods which profit from the abilities of both methods. The main advantage of the passive methods is its no need for the external force for separation. This feature leads to rapid and low-cost separation. Also, the viability of the cells can be preserved admissibly due to the absence of external force. The main examples of passive methods of separation include pinch flow fractionation^4^, deterministic lateral displacement^5^, and employing inertial effect such as dean flow fractionation^6^ and Zweifach–Fung effect^7^. On the other hand, active methods can provide some special advantages over the passive counterparts. Active methods often have higher throughput and separation efficiency than passive ones. However, they need more equipment, and the cells can probably be damaged by the external forces used in these methods. Furthermore, most of the active methods suffer from long residence time and consequently low flow rate because the cells should be sufficiently exposed to the external force field. The conventional active methods of cell separation include electrophoresis^8^, dielectrophoresis^9^, magnetophoresis^10^, acoustophoresis^11^, and optical tweezers^12^. Hybrid methods of separation can be another route for cell separation and sorting. In this way, the combination of active and passive methods proffers higher sensitivity and tenability and enhances the ability for multiplex separation^13^. Moreover, utilizing the advantages of both passive and active methods can lead to higher separation efficiency and can decrease the time and cost of the separation process.

In addition to the method of separation, microfluidic platform is another important issue that can affect the quality of the separation. Rotating platform (also known as ‘Lab-on-a-CD’) is one of the promising microfluidic platforms for conducting biological assays. On this platform, fluid flow is supplied by centrifugal force. So, syringe pumps and all the other external interconnections are eliminated. Also, fluid controlling can be easily done by using simple passive valves^14^. This platform has been employed in the integrated biosensors to achieve a high-sensitivity detection of pathogens^15^ and accelerate gene amplification processes^16,17^. Furthermore, the centrifugal force on a spin unit can be employed to separate particles based on size and density. Morijiri et al*.*^18^ implemented a pinched-flow structure on a rotating disk, where different particles were placed on their own pathways based on size and then separated based on size and density in a peripheral channel due to the effect of centrifugal force. Yeo et al*.*^19^ mounted a spiral microchannel on a rotating disk and successfully separated 200 and 500 nm fluorescent nanoparticles at the outlets. They also utilized this device to extract the microvesicles released from the suspension of MCF-7 and H1975 cell lines. Finally, western blot analysis was conducted to distinct two different microvesicles through checking the CD63 exosomal marker. Compared with the conventional methods, the proposed device required less operation time and extracted the microvesicles at ten folds lower rotational speed. Lee et al*.*^20^ constructed a four-layer on-disk filtration device to separate CTCs spiked in a whole blood sample. The sample was driven towards a track-etched polycarbonate membrane by centrifugal force. The pore size of 8 μm was chosen for the membrane, and MCF-7 cells were trapped on the membrane with an average efficiency of 61% for the spin speed in the range of 600–3600 rpm. To avoid membrane clogging, the authors suggested diluting the sample. They also showed that dilution up to 1:3 ratio has no significant effect on capture efficiency or purity.

Some researchers have employed active methods of separation on the rotating platform to create a more compact and effective device for particle sorting. Kirby et al.^21^ developed a lab-on-a-CD magnetophoretic scheme to separate magnetically-tagged particles and cells from a background population. The target cells were specifically labeled with magnetic beads and deflected towards an on-CD permanent magnet inside the stagnant carrier flow. A similar pattern was employed by Glynn et al*.*^22^ to separate CD4-positive cells (HL60) from different spiked samples. The samples were treated with Dynabeads CD4 magnetic beads, and the specific bindings were just formed between HL60 cells and Dynabeads. The results depicted that more than 80% of the target cells would be isolated from different spiked samples. However, when two types of human cells (HL60 as CD4-positive, and HeLa as CD4-negative) were spiked in the sample, the purity of the isolated target cells would be decreased due to some cell–cell interactions. Chen et al*.*^23^ designed a multistage centrifugal microfluidic device that captures magnetically labeled non-target cells. Capillary valves were employed to connect the device stages, and four ring-shaped magnets were located on the device to maximize the magnetic binding force. To fulfill the aim of each stage, the device was rotated based on a specific rotational protocol in the range of 0–1000 rpm. MCF-7 cells were used as the rare target cells in two different spiked samples with a large number of Jurkat cells and mononuclear cells (MNCs). Separation yields were determined for 10 to 1000 MCF-7/$10^{6}$ Jurkat cells and ~ 47 MCF-7/$10^{6}$ MNCs. The results depicted an average yield of 60 ± 10 for all the experiments. To remove the requirement of the cell labeling process, Shamloo and Besanjideh^24^ suggested utilizing negative magnetophoresis on a rotating disk. The proposed device was reinforced by a dual-purpose permanent magnet that could participate in mixing the sample and ferrofluid and also exert the magnetic repulsive force on the particles. The results depicted that the combination of centrifugal and magnetic force can improve recovery rates for binary and ternary separation of particles.

**Figure 1:**
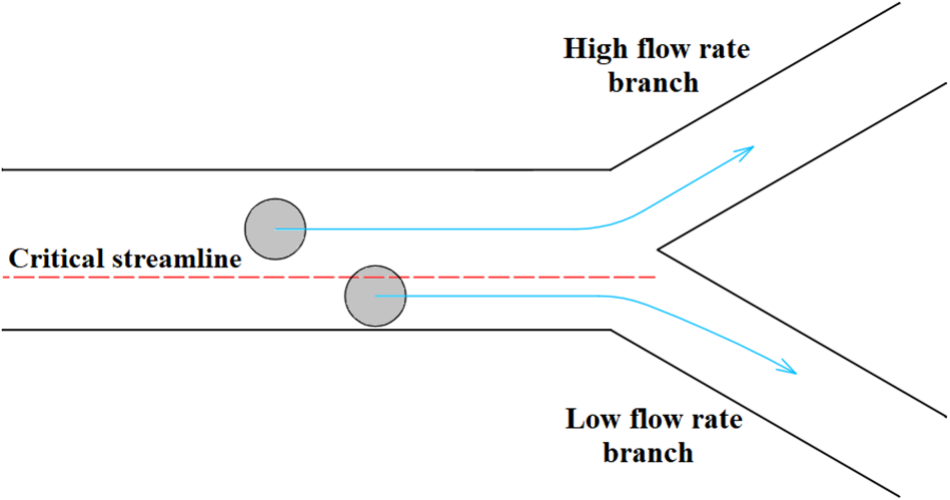
Effect of bifurcation region on separation of particles. Particles placed above the critical streamline are drawn into the high-flow-rate branch while those located under the critical streamline are conducted to the low-flow-rate branch.

The main aim of this study is to introduce a low-cost and simple separation device which can rapidly isolate the rare cells with proper efficiency. Therefore, in the first step, we considered designing a passive separator using just inertial effects on the rotating platform. To this end, combination of a bifurcation region and a contraction–expansion array (CEA) microchannel is considered. Employing a bifurcation region can be considered as a simple and effective passive technique of separation. Based on the bifurcation law, when a suspension of particles flows through a bifurcation region, most of the particles are attracted to the high-flow-rate branch while fewer particles travel into the low-flow-rate branch. This phenomenon is also known as Zweifach–Fung effect and can be explained by the pressure gradient and shear stress distribution applied to the particles^25^. Figure 1 depicts how bifurcation law conducts most of the particles towards the branch with a higher flow rate. If centers of the particles are located above the critical streamline, they are drawn into the high-flow-rate branch. Otherwise, particles are conducted towards the branch with the lower flow rate. It should be noted that the critical streamline is placed near to the low-flow-rate branch, and it approaches more to this branch by increasing the flow rate ratio between two branches. Yang et al.^25^ introduced a microfluidic device for blood plasma separation using bifurcation law. Also, Geng et al.^26^ utilized bifurcation law for separation of plasma from whole blood sample. Their device was also reinforced by centrifugal and diffuser-nozzle effects.

On the other hand, particle focusing and separation can be performed by using inertial hydrodynamic forces. Along a straight microchannel with a parabolic velocity profile, equilibrium positions of particles are determined by balancing wall-induced and shear-induced lift forces. However, in a curved channel, the curvature can induce secondary flow, which exerts Dean drag force to the particles. Balancing lift and Dean drag forces determine the equilibrium positions of particles^27^. De Carlo et al*.*^28^ used an asymmetric curved microchannel for particle focusing. Also, spiral microchannels can employ the Dean drag force to focus and separate the particles^29,30^. It has been observed when particles pass through a straight channel with successive contractions and expansions (CEA microchannel), they experience Dean drag force similar to that exerted on particles into a curved channel with constant cross-section^31,32^. In the entrance of the contraction region, particles are affected by Dean drag forces. However, passing through the contraction region, particles also experience the inertial lift forces^33^. Therefore, a force balancing determines the migration routes of the particles into a CEA microchannel based on the size of particles.

In the second step of this study, the construction of a simple hybrid microseparator was intended so that separation efficiency would be enhanced notably. Therefore, the combination of a CEA microchannel and a magnetophoretic separation technique was considered. Among all conventional active methods, it seems that magnetophoresis is more proper for the separation of rare cells from the blood samples. Magnetic force interacts indirectly with the cells and does not jeopardize their viability^34^. Furthermore, in magnethophoretic assays, the target cells are labeled by magnetic nanoparticles through antigen–antibody complexes. So, in addition to the physical properties, the biological properties of the target cells are involved in separation and consequently, more separation efficiency would be expected.

As deduced from the literature, achieving a simple and effective separation plan is still an unresolved problem for the enrichment of the rare cells. Most of the proposed devices suffer from low purity and recovery rates or have complex designs and impose high costs for mass production. The pivotal reason for this challenge should be sought in the complex hydrodynamic behavior of particle suspensions. Recently, computational models have been widely used to design more effective lab-on-a-chip systems^35,36^. Hence, our plans were optimized based on numerical particle tracing simulations such that the on-disk hydrodynamic phenomena can be utilized for the separation goal. Moreover, experimental results confirm that the proposed plans would be used simply and offer high separation efficiency.

## Theory

### Device description

**Figure 2:**
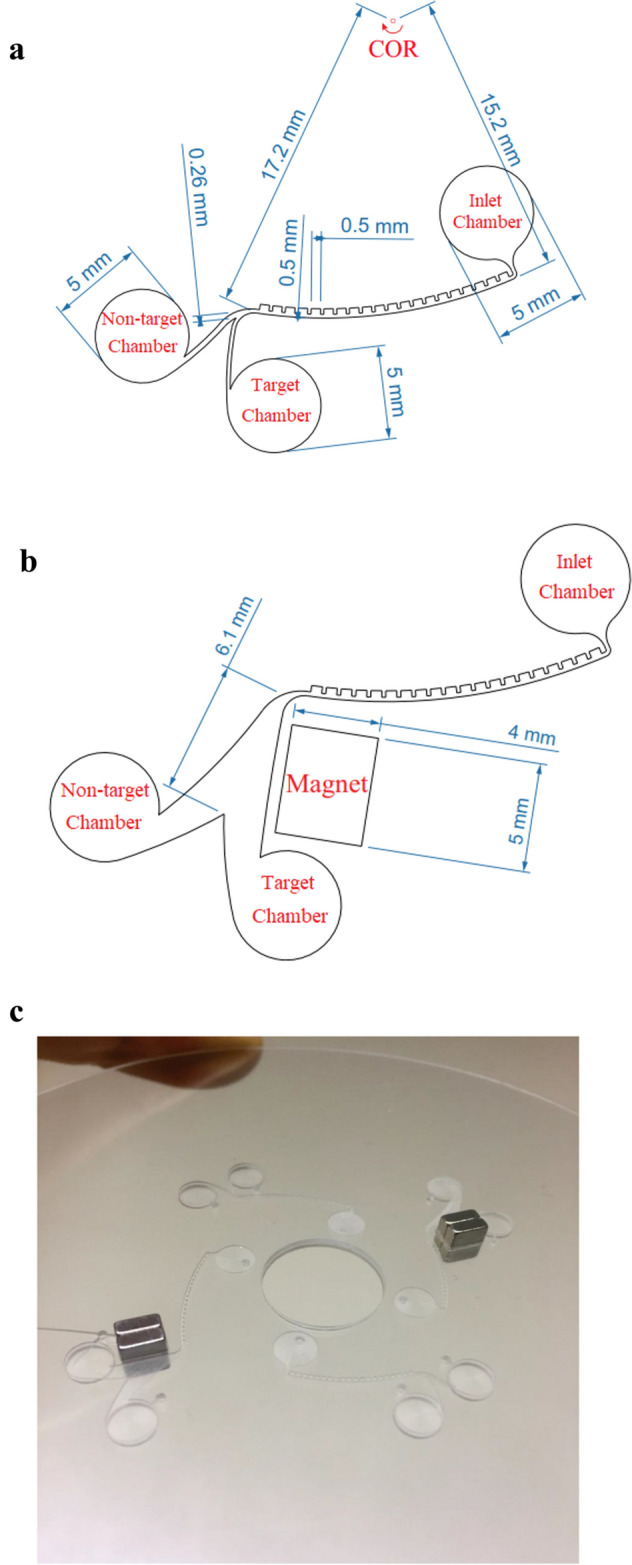
Schematic illustration of the separation device. (**a**) Passive design. (**b**) Hybrid design. (**c**) Device fabricated by CNC micromachining of a plexiglass plate.

Figure 2a shows the passive design of the separation device, which utilizes both inertial hydrodynamic forces and bifurcation law for cell enrichment. This device comprises a peripheral CEA microchannel, which is directly connected to a bifurcation region at its last part. Centrifugal force acts on particles inside the inlet chamber and drives them into the peripheral CEA microchannel, where particles are aligned on their equilibrium paths through force balancing. It has been proven that by utilizing a CEA microchannel on a stationary platform, particles are sorted based on the size by balancing lift and dean drag forces^31^. However, on the rotational platform, centrifugal force, which is a size-dependent force, also acts effectively to separate two different-sized particles. At the end of the CEA microchannel, the fluid flow is divided into two branches of non-equal flow rates to utilize bifurcation phenomena for cell separation. Design of this region was optimized by numerical simulations so that the target cells are placed on streamlines below the critical streamline and conducted to the low-flow-rate branch, which pours into the target chamber. On the other hand, most of the non-target cells are conducted towards the upper side of CEA microchannel by the effect of inertial forces and flow through the high-flow-rate branch, which pours into the non-target chamber. Therefore, two different-sized particles can be separated by employing the inertial effects of CEA microchannel and bifurcation law.

To employ the hybrid design of the separation device, the bifurcation region is replaced with a magnetic region at the end of the CEA channel. Therefore, the magnetically tagged cancer cells would be captured by the permanent magnets. Figure 2b shows the desired hybrid design of separator, which takes advantage of inertial forces and magnetophoretic separation technique for cell enrichment. Figure 2c shows the four-sector centrifugal platform fabricated by CNC micromachining.

### Governing equations

The governing equations of fluid flow consist of continuity and momentum equations of Newtonian and incompressible fluid flow which are expressed as follow:

1 $$ \nabla \cdot \vec{V} = 0 $$

2 $$ \rho \left( {\vec{V} \cdot \nabla } \right)\user2{ }\vec{V} = - \nabla P + \mu \nabla^{2} \vec{V} + \vec{f}_{b} $$

where $\vec{V}$ is the velocity vector (ms^−1^), P is pressure (Pa), and $\rho$ and µ represent density (kg m^−3^) and dynamic viscosity (Nsm^−2^) of fluid, respectively. $\vec{f}_{b}$ denotes the body forces (Nm^−3^), which consists of centrifugal force ($\rho \vec{\omega } \times \left( {\vec{r} \times \vec{\omega }} \right)$), Coriolis force ($2\rho \vec{V} \times \vec{\omega }$), and Euler force ($\rho \overrightarrow {{\dot{\omega }}} \times \vec{r}$). $\vec{\omega }$ and $\vec{r}$ refer to angular velocity and distance from the center of rotation, respectively. It should be noted that Euler force is neglected supposing constant disk rotational speed.

To determine the trajectory of particles, the Lagrangian approach of Newton’s second law of motion is considered as follows:

3 $$ m_{p} \frac{{d^{2} \vec{r}_{p} }}{{dt^{2} }} = \vec{F}_{drag} + \vec{F}_{lift} + \vec{F}_{centrifugal} + \vec{F}_{Coriolis} + \vec{F}_{magnetic} $$

During the numerical simulation of this work, fluid velocity and pressure fields were obtained by simultaneously solving Eqs. (1) and (2). Then, the trajectories of the particles were determined by employing Eq. (3). It should be noted that the magnitude of forces mentioned in this equation is dependent on the particle Reynolds number defined as follows:

4 $$ Re_{p} = \frac{{\rho d_{p} \left| {V - V_{p} } \right|}}{\mu } $$

where $d_{p}$ is the particle diameter and $V$, and $V_{p}$ denote fluid and particle velocities, respectively. Drag force action on a particle with mass of $m_{p}$ can be obtained by applying Stokes drag force as bellow:

5 $$ F_{drag} = \frac{1}{{\tau_{p} }}m_{p} \left( {V - V_{p} } \right) $$

In order to consider the effect of fluid inertia on drag force, $\tau_{p}$ is calculated by Schiller-Naumann correlation as follows^37^:

6 $$ \tau_{p} = \frac{{4\rho_{p} d_{p}^{2} }}{{3\mu C_{D} Re_{p} }} $$

where

7 $$ C_{D} = \frac{24}{{Re_{p} }} \left( {1 + 0.15Re_{p}^{0.678} } \right) $$

Total lift force acting on the particles is the result of shear-induced and wall-induced lift forces which can be calculated by an overall relation suggested by Asmolov^38^ for $Re_{p} < 1:$

8 $$ F_{lift} = \frac{{C_{l} U_{m}^{2} d_{p}^{4} }}{{D_{h}^{2} }} $$

where $C_{l}$ is the lift coefficient, which depends on the location of particles in the cross-section of the channel. $U_{m}$ is the maximum flow velocity, and $D_{h}$ is hydraulic diameter defined as $2wh/\left( {w + h} \right)$ ($w$ and $h$ denotes width and height of channel, respectively).

On the centrifugal platform, particles are affected by centrifugal, Coriolis, and Euler forces. Euler force is often ignored, assuming constant angular velocity. Centrifugal force is exerted on particles radially outwards from the center of rotation, while the Coriolis force acts perpendicular to angular velocity and particle velocity vectors. These forces can be calculated by the following equations:

9 $$ \vec{F}_{centrifugal} = m_{p} \vec{\omega } \times \left( {\vec{r}_{p} \times \vec{\omega }} \right) $$

10 $$ \vec{F}_{Coriolis} = 2m_{p} \vec{V} \times \vec{\omega } $$

By applying magnetophoretic technique of separation, magnetic force is exerted on particles labelled with magnetite nanoparticles. This force can be expressed as^39^:

11 $$ \vec{F}_{magnetic} = \frac{{\chi_{p} - \chi_{m} }}{{2\mu_{0} }} V_{p} \left( {\vec{B}.\nabla \vec{B}} \right) $$

where $$\chi_{p}$$, and $$\chi_{m}$$ are magnetic susceptibilities of particle and surrounding medium, respectively. $$V_{p} $$ is the particle volume. $$\vec{B} $$ denotes magnetic flux density (T), and $$\mu_{0}$$ is permeability of the free space ($$4\pi \times 10^{ - 7} \,\,{\text{Hm}}^{ - 1}$$).

## Experimental setup

### Microfluidic device fabrication

Two desired designs of the microfluidic device were fabricated using CNC micromachining of plexiglass plates. The circular plexiglass plates are 0.6 mm thick with a diameter of 120 mm. A 0.2 mm milling bit was used to carve out the patterns of the device into a plate with a depth of 0.2 mm. A second circular plate of the same size was used to close and finalize the microfluidic designs. Vents and sample loading port on both plans were drilled as through holes on this second plate using the same milling bit. Afterward, the pair of plates were bonded to each other using a double-sided pressure-sensitive adhesive (PSA). To reduce the surface roughness of the microchannels, the patterned surface was polished with acetone as a weak solvent of plexiglass. To this end, the method of Ogilvie et al*.*^40^ was used. Based on this method, the acetone container is placed inside a steam bath, and due to the low evaporation point of acetone (55 °C), acetone is vaporized and sprayed onto the plexiglass plate to smooth the machined surface.

The final fabricated centrifugal platform is depicted in Fig. 2c, which consists of two passive and two hybrid designs of the separator. As is shown in this Figure, the hybrid designs are supported with a stack of two NdFeB permanent magnets at the downstream of the CEA channel. Finally, to operate the microfluidic device, the disk was mounted on a rotational apparatus, where the desired rotational speed is adjusted through a PID controller and measured by an optical sensor. Figure S1 depicts a schematic of the electronic circuit of the rotational apparatus.

### Cell counting

To detect the target cells from non-target ones, cell staining method was used. According to this method, the target cells were stained with DAPI fluorescent dye, and two identical optical and fluorescence microscopy images were provided from the samples collected from each outlet chamber. Figure S2 illustrates both optical and fluorescent images taken from a part of the sample extracted from the target chamber of the passive separation device. Both stained and unstained cells can be seen in the optical image while in the fluorescent image, just stained cells are observed. Thereby, the target cells can be distinguished from the population of the non-target cells. To quantify the cell numbers, a Python image processing library was used. The stained cells of each outlet were counted via the fluorescent image while the number of unstained cells can be determined by counting the cells observed in the optical image and subtracting from the stained cell numbers.

### Synthesis of nanoparticles and cell-nanoparticle binding

To exploit the hybrid plan, the target cells (MCF-7 cells) should be labelled with magnetic nanoparticles. Therefore, Magnetite (${\text{Fe}}_{3} {\text{O}}_{4} )$ nanoparticles were prepared through the coprecipitation method^41,42^ and then attached to the target cells via antigen–antibody complexes.

In order to synthesis the magnetite nanoparticles, Fe (II) and Fe (III) salts are separately dissolved in hydrochloric acid at 80 °C. Then, these solutions are mixed to produce ${\text{Fe}}^{2 + }$ and ${\text{Fe}}^{3 + }$. Finally, by adding an ammonia solution, magnetite is formed as a black precipitate. After drying this sediments, magnetite nanoparticles are obtained. Figure S3 shows magnetite during the drying process and a microscopic image of magnetite nanoparticles with an average diameter of 20 nm.

**Figure 3:**
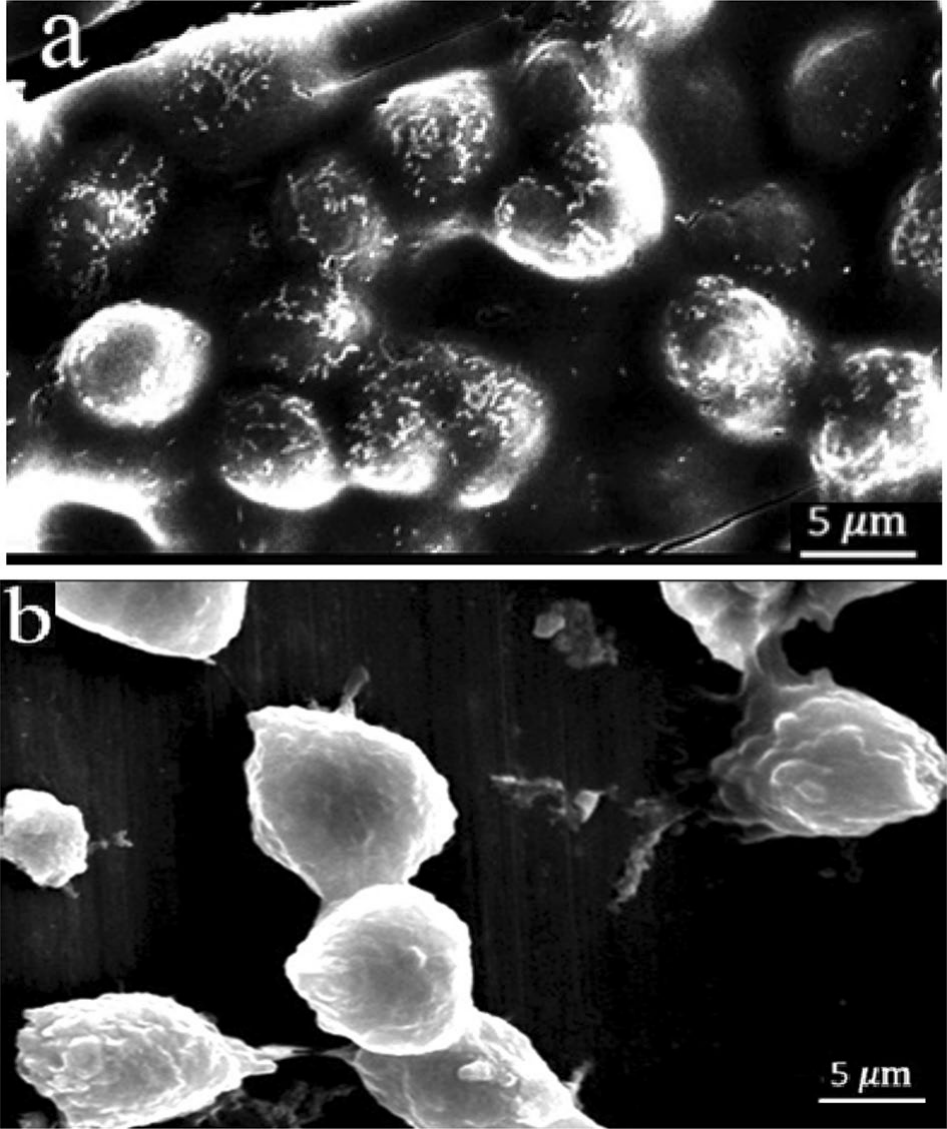
Electron microscopy images of the target and non-target cells in the vicinity of magnetite nanoparticles. (**a**) Binding of magnetite nanoparticles and MCF-7 cells through specific EP-CAM antibodies. (**b**) Nanoparticles are not bonded to L929 cells.

By adding some interfering substances, magnetite nanoparticles can be bound to the EP-CAM antibodies, which in turn, specifically bind to the antigens on MCF-7 cells. Arginine, as an α-amino acid with two amine and carboxyl groups, can trap magnetite nanoparticles through a shell-core binding so that the carboxyl groups are located outwards and can be bound to the amine group of the antibodies. To active the functional groups and forming nanoparticle-antibody conjugation, 1-Ethyl-3-dimethylaminopropyl (EDC) and N-Hydroxysuccinimide (NHS) were used. Figure 3 depicts the specific binding of magnetite nanoparticles to the MCF-7 cells. As is seen in Fig. 3a, magnetite nanoparticles are aggregated around the MCF-7 cell membranes. This type of binding is specifically formed between EP-CAM antibodies and MCF-7 cells. It should be noted that non-target cells (L929 cells in this study) are not affected by magnetite nanoparticles, as is seen in Fig. 3b.

## Results and discussion

### Simulation results

Since the experimental optimization of microfluidic devices is always a tedious and time-consuming process, numerical simulations can be a good alternative to achieve the best design of these systems. In this work, simulations were performed by the finite element method. Fluid velocity and pressure fields were determined by solving the governing equations of fluid flow. Afterward, to obtain the trajectories of particles, Newton’s second law of motion was applied to the particles by the Lagrangian approach.

**Figure 4:**
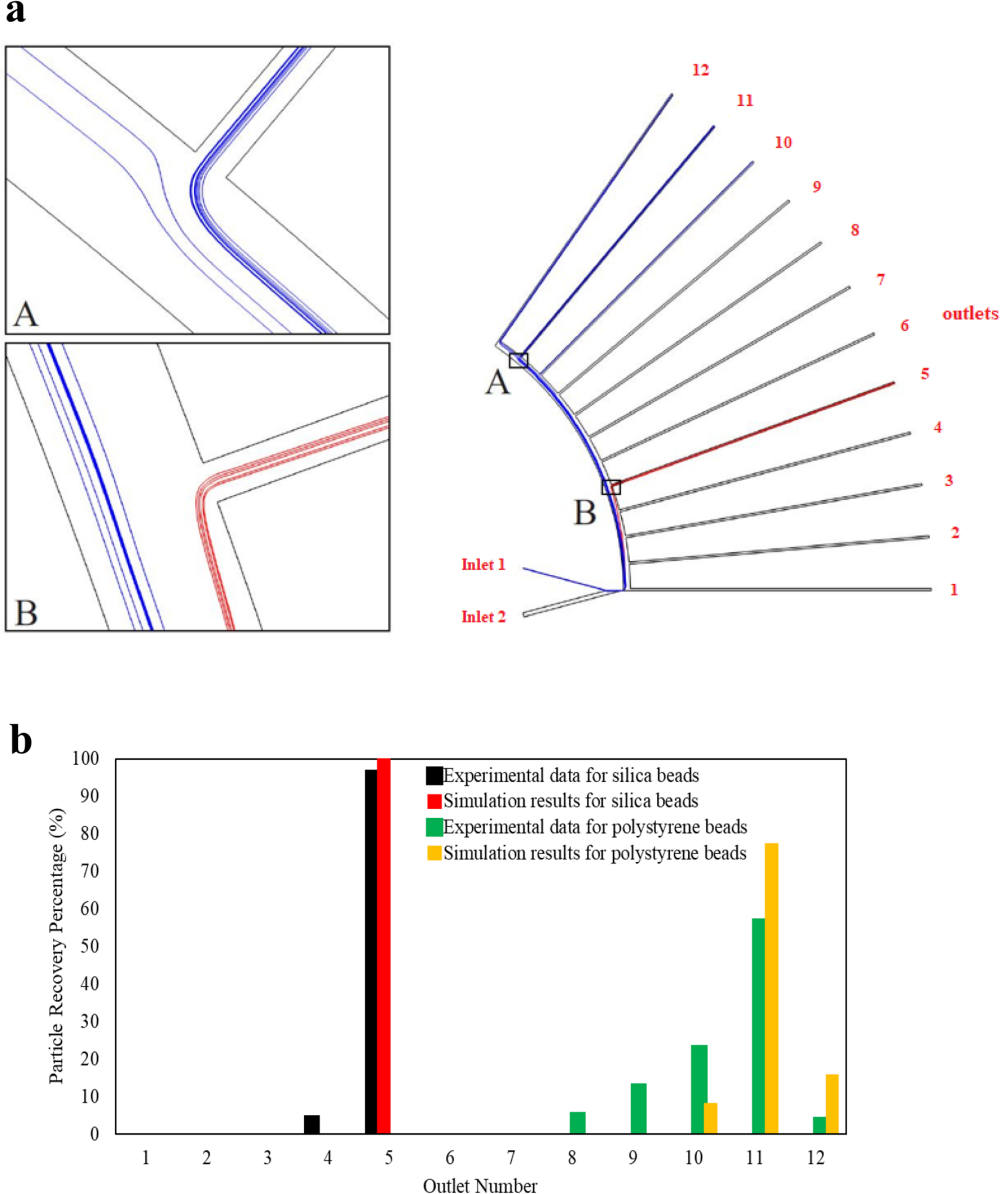
Comparison of simulation and experimental results of Morijiri et al*.*^18^ at the rotational speed of 750 rpm. (**a**) Simulated trajectories of two particles with different sizes and densities (red: silica beads, and blue: polystyrene beads). (**b**) Recovery rates obtained by the numerical methods are compared with the experiments.

As the first step of the numerical investigation, simulation results of a centrifugal microfluidic separator were compared with experiments conducted by Morijiri et al*.*^18^. Figure 4a shows this Lab-on-a-CD device and the simulated trajectories of two different-sized silica and polystyrene particles at the rotational speed of 750 rpm. Particles are entered through inlet 1, and the sheath flow of inlet 2 focuses them onto the upper sidewall in the pinched segment. Particles are arranged on different streamlines based on their sizes and then are sorted by centrifugal force based on size and density. According to the simulations, all the silica beads ($\rho = 2.0$ g cm^−3^, *d* = 5 μm) leave the device through outlet 5, and the polystyrene beads ($\rho = 1.05$ g cm^−3^, *d* = 3 μm) are collected from outlets 10–12. Figure 4b compares the recovery rates of simulation results with that of the experiment. As is evident from this figure, numerical simulations are in good agreement with experiments.

To ensure that the results are independent of the grid size, a grid independence study was conducted. Several grid densities were constructed, and velocity fields and particle recovery rates were investigated. It was found that an unstructured tetrahedral grid with a mean characteristic size of 23 μm is proper for the passive design of separator. Also, a similar grid with a mean characteristic size of 36 μm was found proper for the fluid domain of hybrid design. A summary of the mesh independence study is presented in Table S1 and Figures S4 and S5. Moreover, Figures S6 and S7 show the selected grids for the passive design and the end part of the hybrid design, respectively.

To exploit the best effect of CEA microchannel and bifurcation phenomena on particle separation, several microchannel designs and disk rotational speeds were examined. Also, for the special case of the hybrid plan, the design of the magnetic region and magnet’s strength were optimized to achieve the best performance of separation. MCF-7 breast cancer cells and mouse fibroblast L929 cells were chosen as the target and non-target cells, respectively. Also, the cell ratio in the sample was assumed to be 1:10 as MCF-7s/L929s. Based on experimental measurements, the average size of MCF-7 and L929 cells were considered 23 µm and 14 µm, respectively. Besides, physical properties of the sample and magnet are presented in Table 1. To evaluate the performance of separation device, recovery rate and purity are introduced as two determinant parameters. Recovery rate is defined as the ratio of the number of a specific cell collected from a chamber over the whole number of the same cell. Also, purity is defined as the ratio of the number of a specific cell recovered from a chamber over the whole number of cells existing in the same chamber.

**Table 1:** Physical properties considered for simulations.

Fluid viscosity (µ)	1.002 × 10^–3^ Nsm^−2^
Fluid density (ρ)	100 kg m^−3^
Particle density ($\rho_{p}$)	1050 kg m^−3^
Magnet’s strength ($B$)	0.34 T
Relative permeability of magnet	3.6

**Figure 5:**
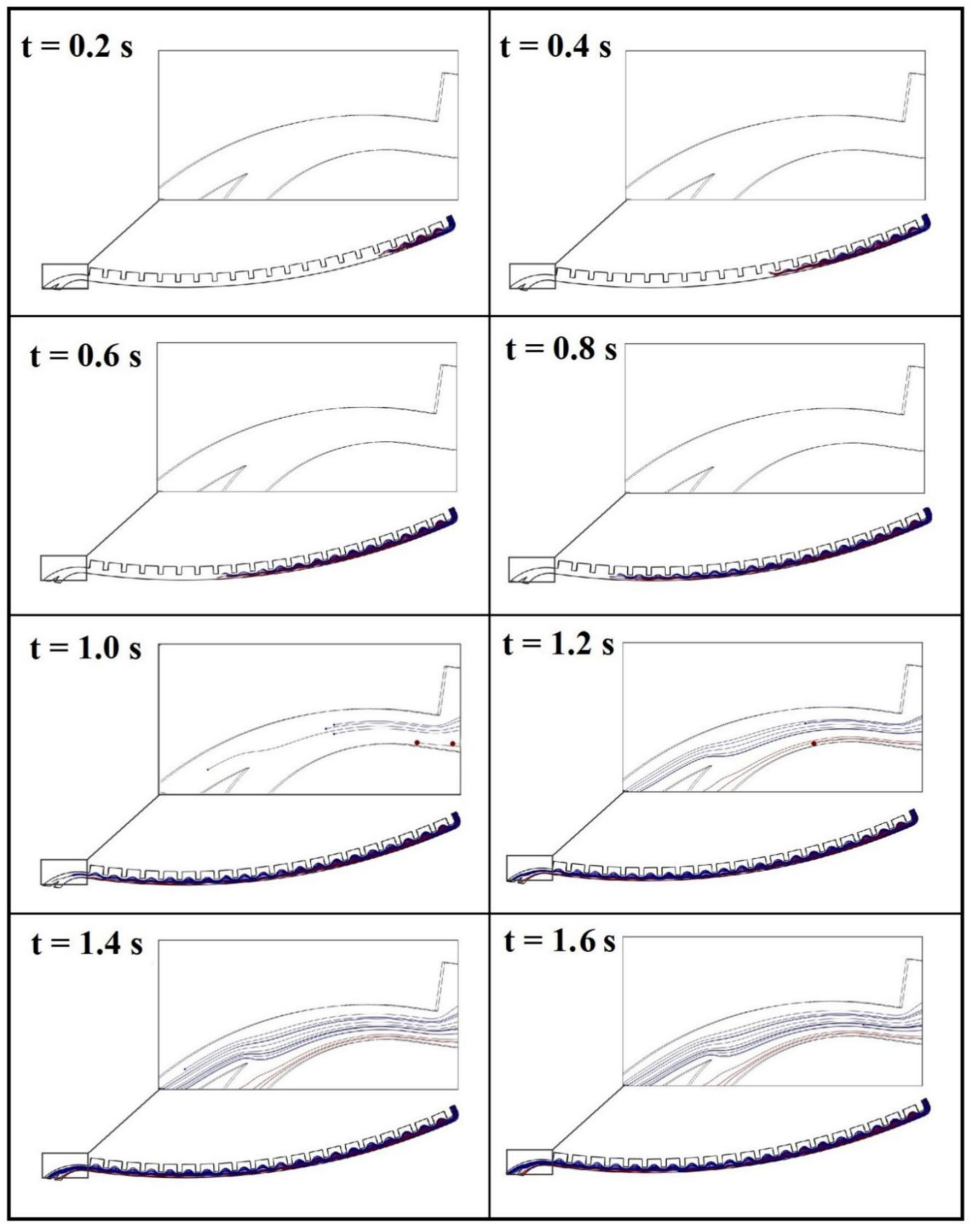
Simulated trajectories of 23 µm-particles (as MCF-7s) in red lines and 14 µm-particles (as L929s) in blue lines by using passive design of separator at the rotational speed of 2400 rpm.

Simulation results of the passive design of separator show that a CEA microchannel with 20 contraction–expansion steps which is connected to a bifurcation region would be able to conduct two different-sized particles (14 and 23 µm) in approximately two distinct pathways. This ability can also be guaranteed for relatively a wide range of rotational speeds (900–3000 rpm). However, the best performance of the separation device could be achieved at 2400 rpm. In this rotational speed, 100% of MCF-7s are collected from the target chamber with a purity of 78%. Figure S8 shows the contour plots of the fluid velocity for the passive design at disk rotational speed of 2400 rpm. Figure 5 depicts the trajectories of particles at different times with intervals of 0.2 s. In this figure, trajectories of the target particles (MCF-7 cells) and non-target particles (L929 cells) are shown in red and blue lines, respectively. As illustrated in this figure, after successive flow contractions and expansions, the target particles are deflected towards the outer streamlines (respect to the center of rotation). Subsequently, these particles pass underneath the critical streamline and are entered into the low-flow-rate branch. On the other hand, non-target particles are located on inner streamlines that pass above the critical streamline and conducted inside the high-flow-rate branch. Therefore, the target particles can be enriched through a contraction–expansion array and bifurcation phenomena.

In the hybrid design, a CEA microchannel similar to that of passive design is employed to take apart the directions of two different-sized particles. However, the target particles are assumed to be labeled with paramagnetic nanoparticles and experience a magnetic field at the end part of the separation device. In this way, the target particles are attracted to the target chamber through the magnetic force, while the non-target particles move along their paths towards the non-target chamber.

**Figure 6:**
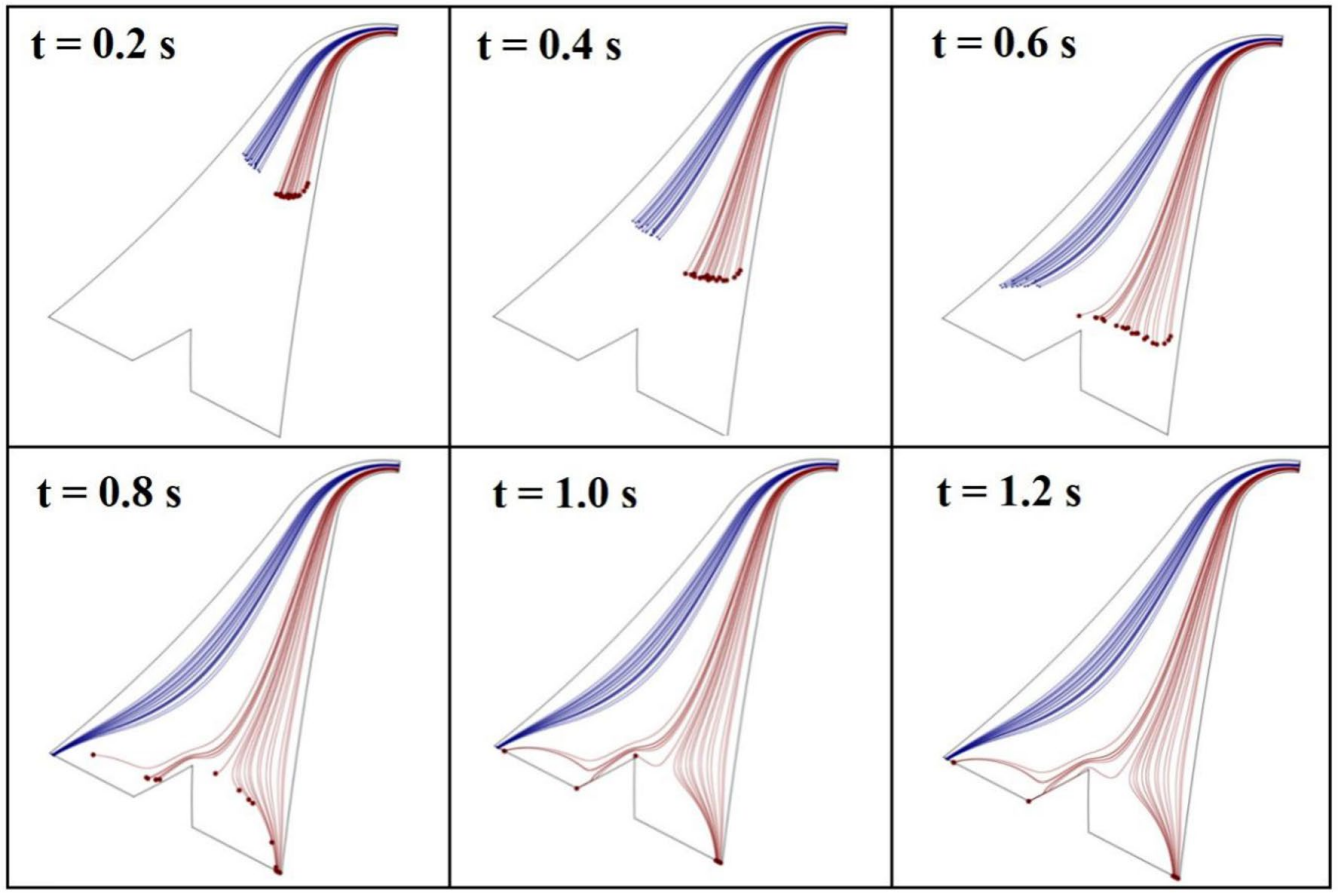
Simulated trajectories of 23 µm-particles (as MCF-7s) in red lines and 14 µm-particles (as L929s) in blue lines at the end of the hybrid design of separator at the rotational speed of 2100 rpm.

To avoid the huge memory usage of hybrid plan simulations, just the magnetic region of this plan was investigated and the boundary conditions at the inlet of this region were considered as those which calculated for this section of passive plan. To study the effect of disk rotational speed on separation efficiency, a wide range of rotational speed (900–3000 rpm) was considered. As an example, the trajectories of particles at the rotational speed of 2100 rpm is depicted in Fig. 6 at different times with intervals of 0.2 s. In this situation, 70% of the target cells are collected from the target chamber with purity of close to 100%. Simulation results predict that the best performance of the hybrid plan can be achieved at 1500 rpm so that 100% of the target cells are recovered from the target chamber with 100% purity. Based on simulation results of passive and hybrid devices, we selected the proper test range of rotational speed for each of them to achieve high recovery rate of target cells and low recovery rate of non-target cells, simultaneously.

### Experimental results

**Figure 7:**
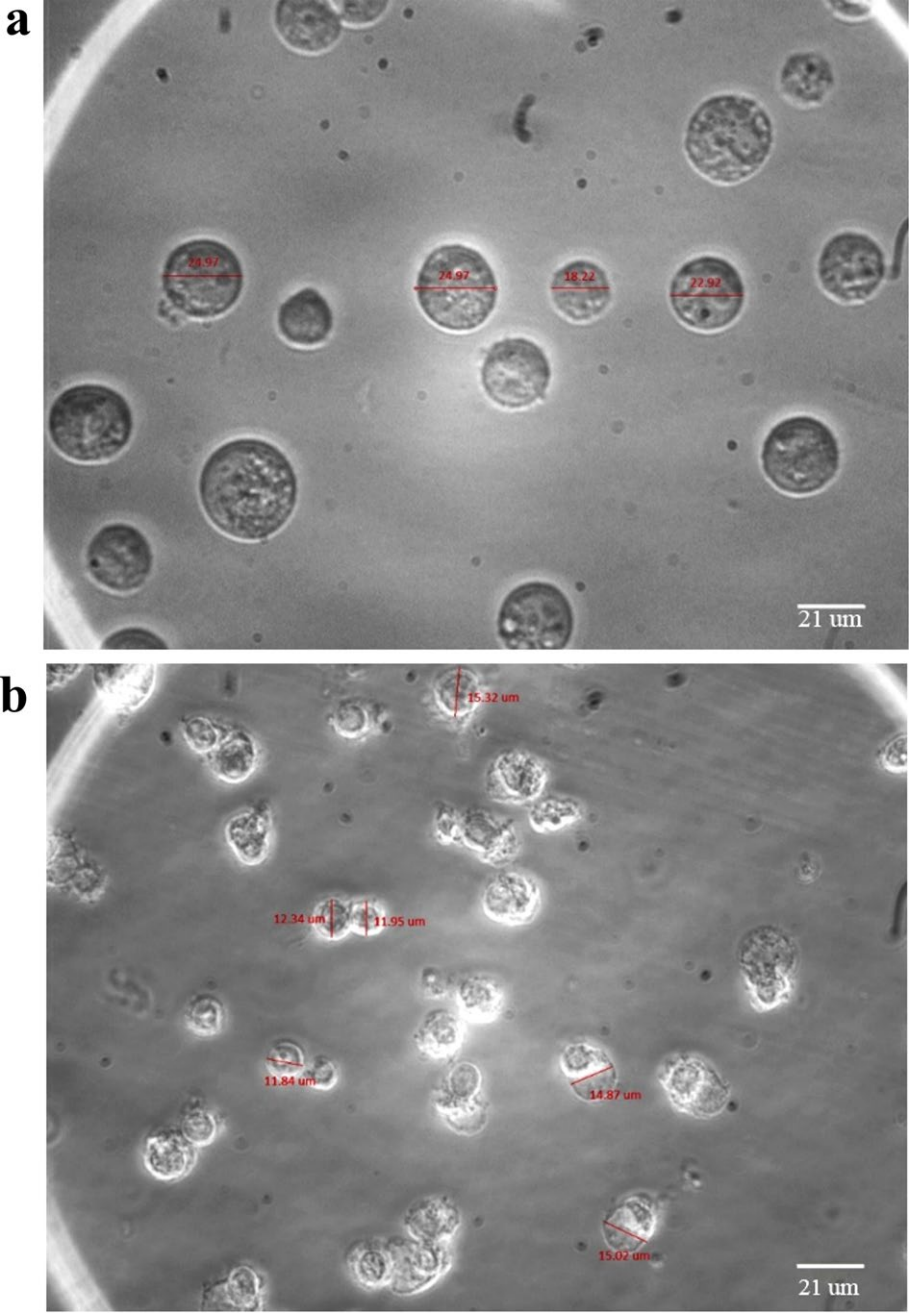
Size distribution of target and non-target cells. (**a**) MCF-7 cells with a mean diameter of 23 µm and (**b**) L929 cells with a mean diameter of 14 µm.

MCF-7 cells as target cancer cells, and L929 mouse fibroblasts as non-target cells were cultured at 37 °C, 5% ${\text{CO}}_{2}$, and 95% relative humidity. Figure 7 shows size distribution of MCF-7 and L929 cells with mean diameters of 23 µm and 14 µm, respectively. The target cells were stained with DAPI fluorescent dye and then rinsed with Phosphate-buffered saline (PBS) solution. The final sample was prepared by suspending the cells in 15 ml PBS including 2% fetal bovine serum (FBS). Meanwhile, 4% glutaraldehyde was added into sample to prevent cell aggregation. It should be recalled that to employ the hybrid plan, magnetite nanoparticles were bound to the target cells using the method described previously, and glutaraldehyde was not used in sample due to the its adverse effect on antigen–antibody interactions.

Before using the separation device, microchannels were filled with PBS to reduce the effects of surface tension. The spike sample of MCF7 and L-929 cells was prepared for the tests with a cell ratio of 1:10 and the overall cell count of ~ 5000 cells per microliter of PBS buffer. After the injection of 2 µl of the spike sample in the inlet chamber, each test was run for one minute and the tests were replicated 5 times for each intended disk rotational speed. At the end of each trial, the outlet chambers were drained separately into two petri dishes, where their contents were analyzed to count the cell numbers by the method previously discussed. It should be mentioned that at the end of each test, microchannels were rinsed with PBS and trypsin to ensure that all the cells are detached from the channel surfaces.

**Figure 8:**
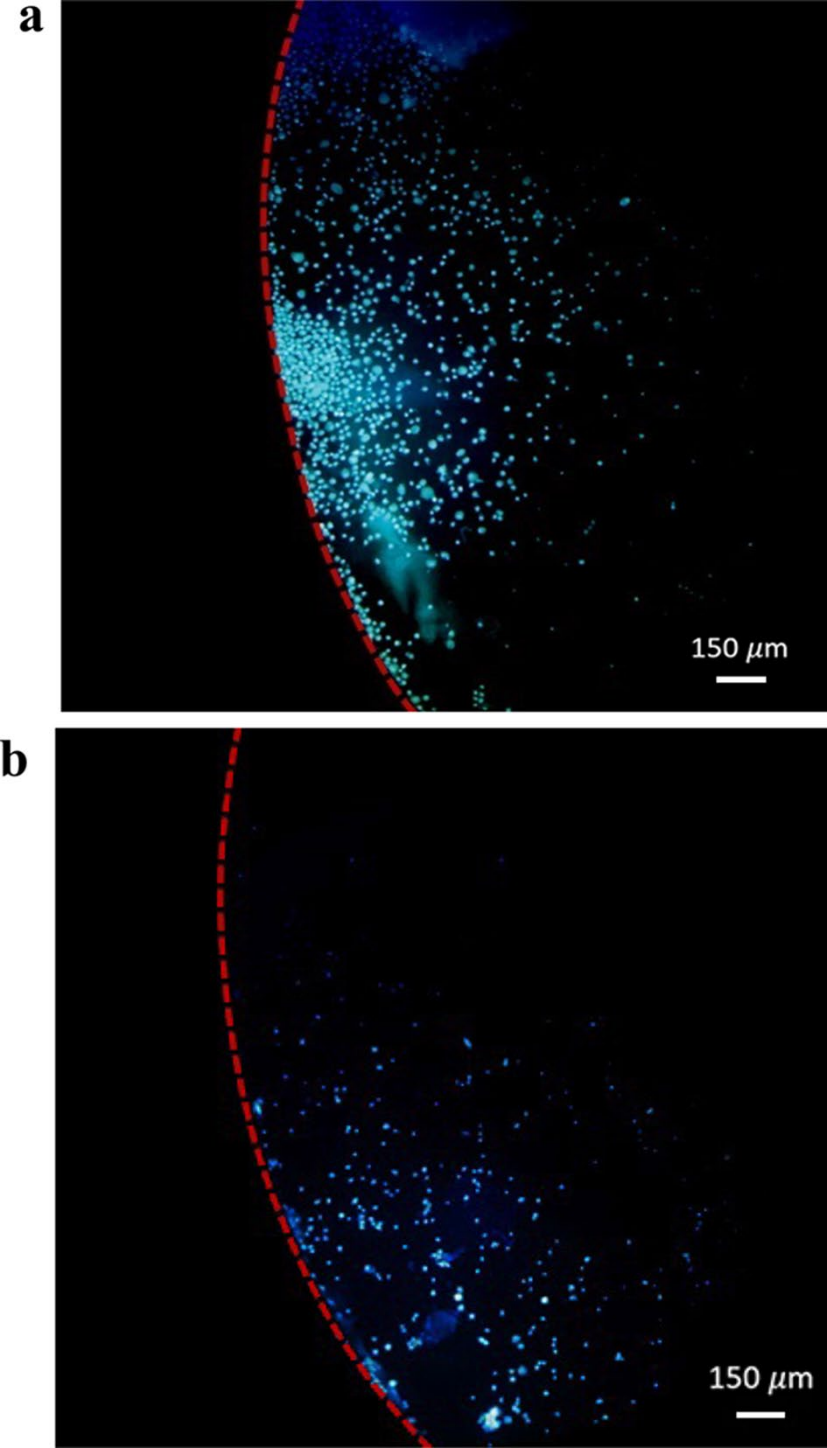
Fluorescent images of MCF-7 cells captured by the passive centrifugal separator at the rotational speed of 2100 rpm. (**a**) Target chamber. (**b**) Non-target chamber.

**Figure 9:**
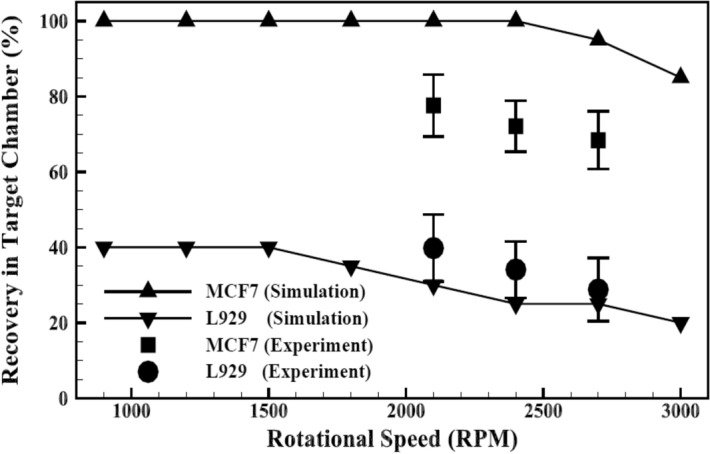
Numerical and experimental recovery rates of MCF-7 and L929 cells from the target chamber of the passive design of separator.

In order to experimentally investigate the passive design of separation device, the device was tested at three different rotational speeds of 2100, 2400, and 2700 rpm. Figure 8 shows the fluorescent images of MCF-7 cells captured in target and non-target chambers at the rotational speed of 2100 rpm. To summarize all the results obtained from the passive design of separator, Fig. 9 represents both numerical and experimental recovery rates of MCF-7 and L929 cells from the target chamber. Based on experiments, the maximum recovery rate of target cells in the passive design of separator can be achieved at the rotational speed of 2100 rpm. This result is relatively consistent with the results observed from simulation, which predicted the best performance of the passive plan at the rotational speed of 2400 rpm. In the rotational speed of 2100 rpm, 76% of MCF-7 cells were recovered from target chamber. Nevertheless, about 40% of L929 cells were also collected into this chamber. Considering the trends of simulation results suggests that testing the device at lower rotational speeds would cause an increase in the recovery rate of non-target cells that is not desirable as it adversely affects the target cell purity.

**Figure 10:**
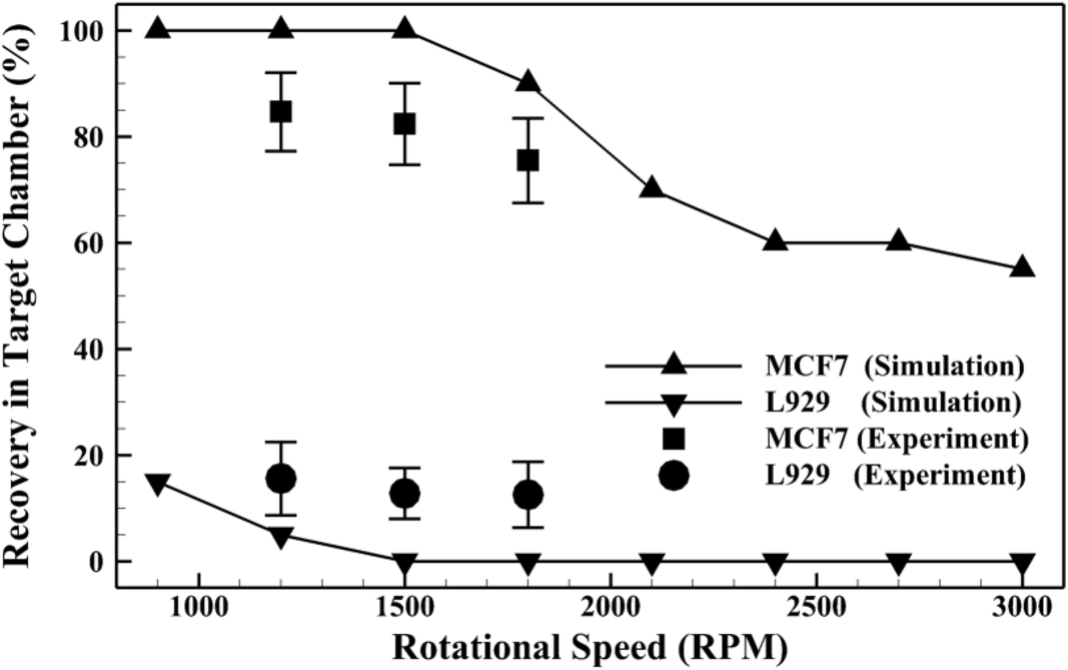
Numerical and experimental recovery rates of MCF-7 and L929 cells from the target chamber of the hybrid design of separator.

Finally, hybrid design of separation device was tested experimentally. To this end, device performance was evaluated at three different rotational speeds of 1200, 1500, and 1800 rpm. The respective recovery rates of MCF-7 and L929 cells from the target chamber are presented in Fig. 10. Meanwhile, the simulation results of the hybrid plan are illustrated and compared with experimental data in this figure. It is obvious that experimental and numerical results are in good agreement. Furthermore, the maximum recovery rate of target cells is predicted at the rotational speed of 1200 rpm, where 85% of MCF-7 and 18% of L929 cells are collected into the target chamber. This result conforms to the findings from simulations at 1200 rpm, where all the target cells were recovered into the target chamber with 100% purity.

A comparison between two studied plans of separation device shows that the hybrid design of separator is able to separate more percentage of the target cells with higher purity. On the other hand, the passive design of separator is easily operated without the time-consuming process of cell labeling and imposing additional costs. Consequently, there is a tradeoff between accuracy and easiness of separation method to choose the desired design of separator.

## Conclusion

In this study, two passive and hybrid centrifugal designs of a separator device were investigated for enrichment of the rare cancer cells. The passive design utilizes the advantages of CEA microchannel and bifurcation to separate cancer cells from the population of non-target cells. However, in hybrid design, the CEA microchannel is directly connected to a magnetic region, where a magnetophoretic technique is utilized to separate the magnetically labelled target cells from the non-target ones. Numerical simulations were carried out to determine the optimized geometries and disk rotational speeds for both passive and hybrid designs. Afterward, the optimized designs were fabricated through CNC micromachining of plexiglass plates and tested to separate MCF-7 cells as target cells from the population of mouse L929 cells as non-target cells. To employ the hybrid plan, magnetite nanoparticles were bound to MCF-7 cells through the specific EP-CAM antibodies. Experiment results indicated that by utilizing the passive design of the separator at 2100 rpm, 76% of MCF-7 cells were recovered from the target chamber while 40% of L929 cells were also collected from this chamber. Additionally, the hybrid design was able to collect 85% of MCF-7 cells and only 18% of L929 into the target chamber at the rotational speed of 1200 rpm. As is evident from the experiments conducted in the reported rotational speeds, the hybrid design of separator is able to simultaneously offer a higher recovery rate and purity for cell enrichment at the optimal rotational speeds suggested by the simulation results. Nevertheless, the passive design can be a convenient choice for rapid and admissible enrichment of cancer cells with no need for grueling and time-consuming process of cell labeling.

## Supplementary Information

Supplementary Information
