# Supplementary material for: Cancer cell enrichment on a centrifugal microfluidic platform using hydrodynamic and magnetophoretic techniques
Source: Sci Rep. 2021 Jan 21;11:1939. doi: 10.1038/s41598-021-81661-2 (PMC7820336; doi:10.1038/s41598-021-81661-2)
Supplement: Supplementary file 1 — Supplementary Information [file 41598_2021_81661_MOESM1_ESM.docx]

**Supplementary information for**

**Cancer Cell Enrichment on a Centrifugal Microfluidic Platform Using Hydrodynamic and Magnetophoretic Techniques**

**Amir Shamloo^1*^, Amin Naghdloo^1^, Mohsen Besanjideh^1^**

*^1^Department of Mechanical Engineering, Sharif University of Technology, Tehran, Iran*

*
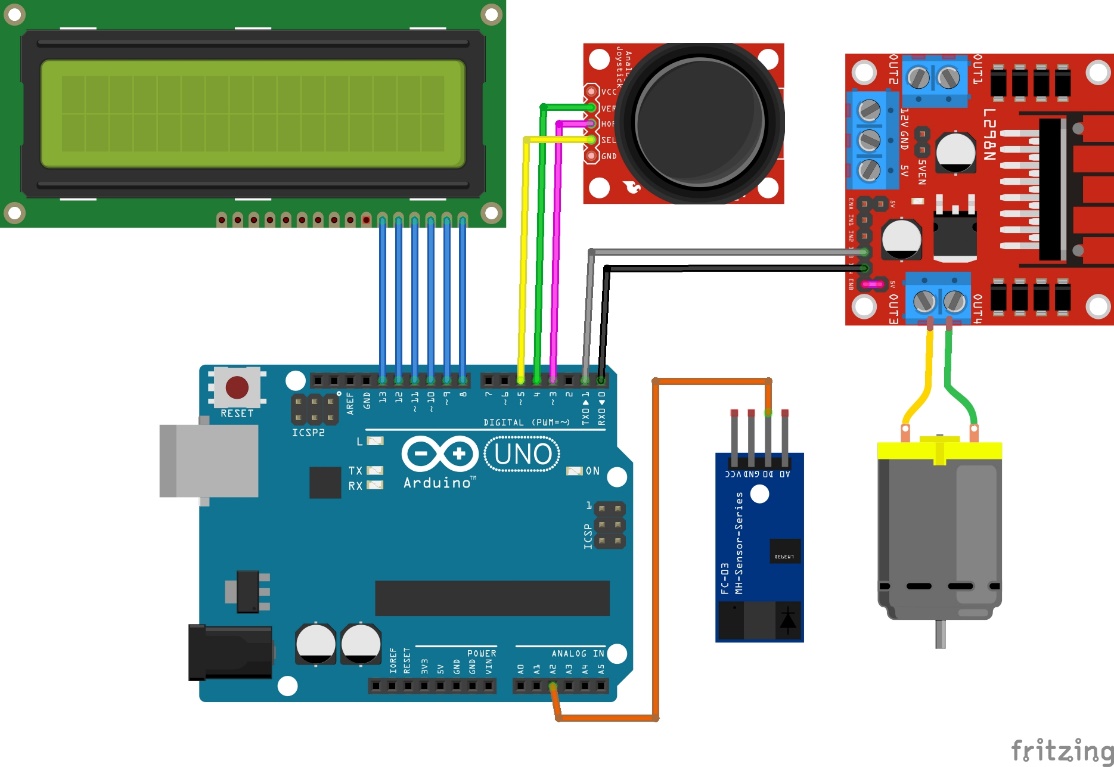
*

**Supplementary Figure S1.** schematic of the electronic circuit of the rotational apparatus [Used by permission of Fritzing software company under CC-BY-SA 3.0, from Amin Naghdloo (2020)].


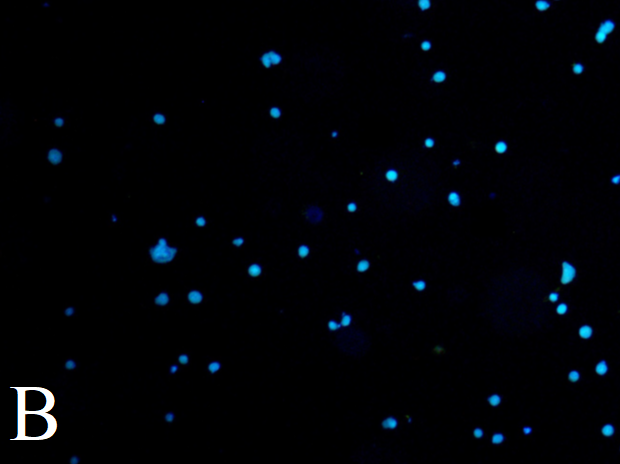

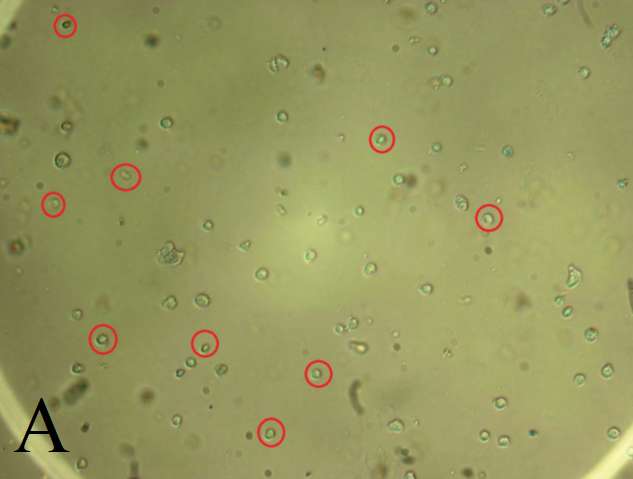


**Supplementary Figure S2.** (A) optical and (B) fluorescent images from a part of the sample extracted from the target chamber of the passive separation device. Stained cells are depicted in both images, while the unstained cells are just seen in the optical image (denoted by red circles).


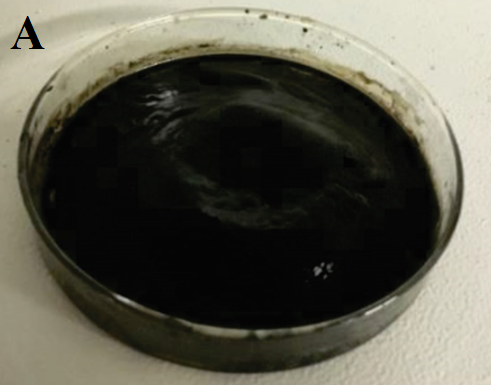

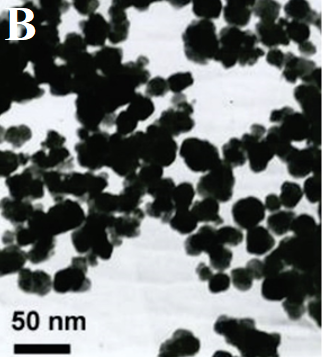


**Supplementary Figure S3.** (A) magnetite sediments during the drying process. (B) microscopic images of magnetite nanoparticles.

**Supplementary Table S1.** Numerical results of the recovery rates from the target chamber of passive and Hybrid separators corresponding to different grid densities at 1800 rpm.

| **Design** | **Case number** | **Mesh average size (𝜇m)** | **Recovery rate from the target chamber(%)** | |
| --- | --- | --- | --- | --- |
|  |  |  | **MCF-7** | **L929** |
| **Passive** | 1 | 48 | 94 | 31 |
|  | 2 | 35 | 100 | 35 |
|  | 3 | 23 | 100 | 35 |
|  | 4 | 14 | 100 | 35 |
| **Hybrid** | 1 | 76 | 100 | 8 |
|  | 2 | 51 | 90 | 8 |
|  | 3 | 36 | 90 | 0 |
|  | 4 | 24 | 90 | 0 |


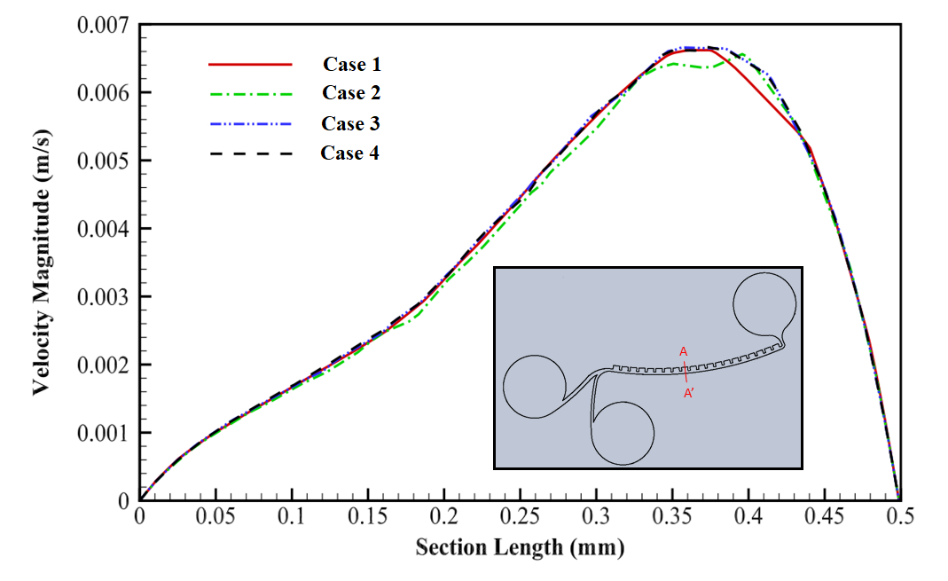


**Supplementary Figure S4.** Velocity profiles at section A-A’ from the passive design of separator corresponding to different grid densities described in Table S1.


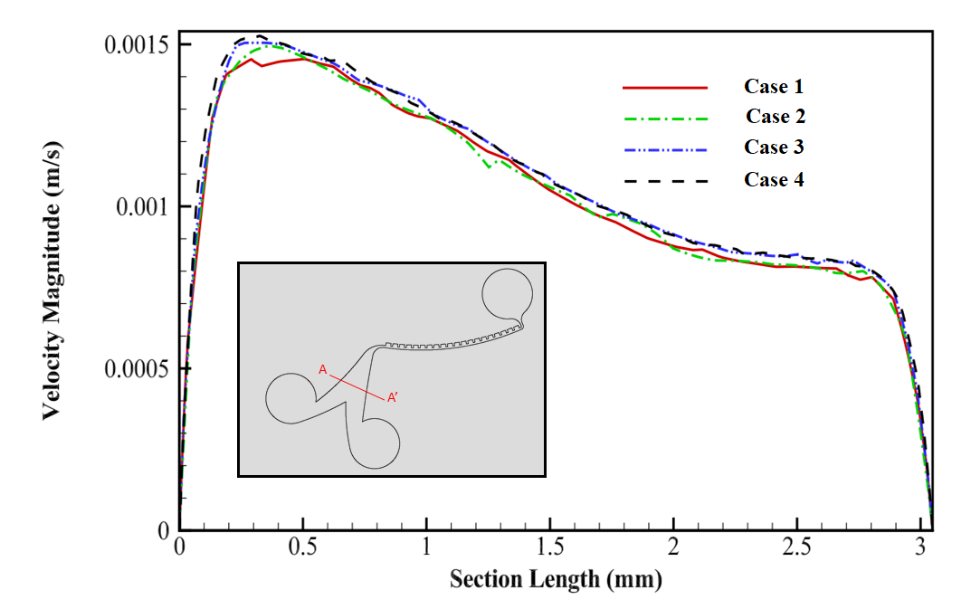


**Supplementary Figure S5.** Velocity profiles at section A-A’ from the hybrid design of separator corresponding to different grid densities described in Table S1.


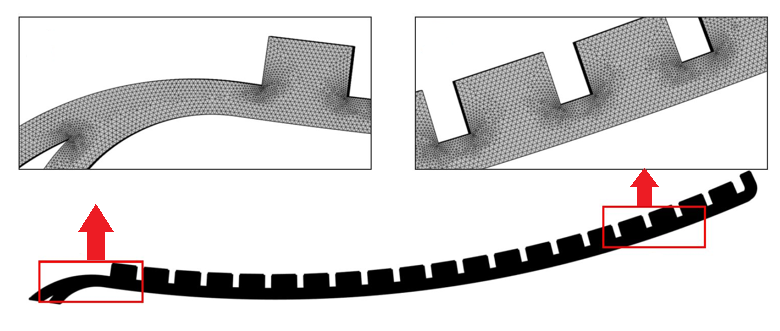


**Supplementary Figure S6.** The grid constructed for passive design of separation device. The red observation windows are magnified for a better view.


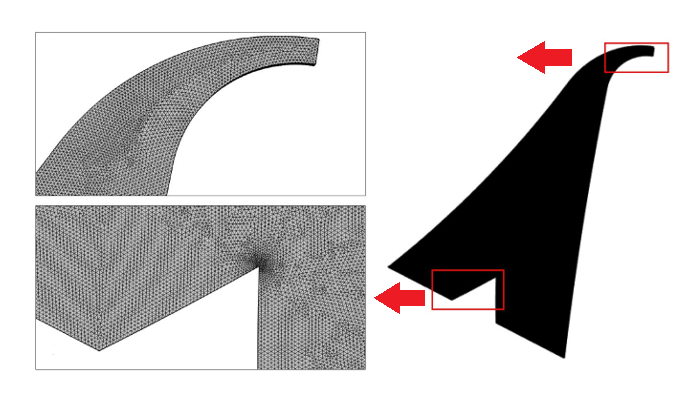


**Supplementary Figure S7.** The grid constructed at the end part of hybrid design of separation device. The red observation windows are magnified for a better view.


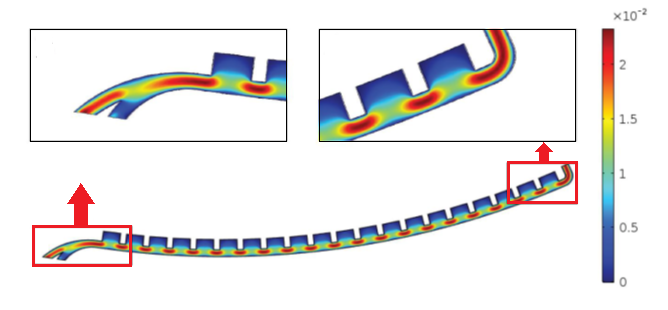


**Supplementary Figure S8.** Contour plots of the fluid velocity ($ms^{-1}$) for the passive design of separation device at rotational speed of 2400 rpm. The red observation windows are magnified for a better view.
